# Shift from complementarity to facilitation on P uptake by intercropped wheat neighboring with faba bean when available soil P is depleted

**DOI:** 10.1038/srep18663

**Published:** 2016-01-05

**Authors:** Chunjie Li, Yan Dong, Haigang Li, Jianbo Shen, Fusuo Zhang

**Affiliations:** 1 Center for Resources, Environment and Food Security (CREFS), China Agricultural University, Beijing, 100193, China; 2 College of Resources and Environment, Yunnan Agricultural University, Kunming, 650201, China

## Abstract

Rhizosphere processes stimulate overyielding and facilitative phosphorus (P) uptake in cereal/legume intercropping systems. However, little is known about when and how rhizosphere alteration of legumes plays a role in improving P uptake by cereals. Wheat was grown isolated, monocropped or intercropped with faba bean in pots with low-P soil. The biomass, P content, carboxylates and phosphatases activity were measured in 15 destructive samplings. Intraspecific competition of the biomass and P uptake of monocropped wheat was not significant before 40 and 36 days after sowing (DAS), whereas there was interspecific competition of biomass of intercropped wheat before 66 DAS. However, afterwards, the increments of the biomass and P uptake of the intercropped wheat were 1.3–1.9 and 1.9–2.3 times of increment of monocropped wheat. Meanwhile, the concentrations of malate and citrate and the acid phosphatase activity in the rhizospheres of intercropped wheat were significantly increased, which suggested that wheat/faba bean intercropping is efficient in P utilization due to complementary P uptake in the early growth stage and the positive interactions of the rhizosphere processes when the soil P was depleted.

Phosphorus (P) is an essential nutrient for all living organisms and is one of the major yield-limiting nutrients in many agricultural systems^1^, because the bioavailability of P in most soils is low^2^. To address this issue, famers apply fertilizers with high P solubility to increase crop yield. However, plants can usually take up only 15-20% of the added P in the growing season^3^, and the remaining P is converted to less available forms for crops^4^. Thus, a substantial part of the added P accumulates in the soil as residual P, contributing to the plant-available P pool for crop production^5^. Many plant species have strategies to acquire the less-soluble phosphate bound in the soil, such as rhizosphere acidification, carboxylate exudation and secretion of phosphatase^2^,^6^,^7^,^8^. These strategies mobilize P for the respective plant, and may also facilitate P uptake by another plant species, provided that that roots are in each other’s vicinity.

Intercropping, i.e., simultaneous cultivation of two or more crop species in the same field^9^, has a long history and is commonly used in China. Cereal/legume intercropping is an effective way to improve plant P uptake from a low P soil through species interactions in the rhizosphere^10^. For example, in one field study, total P acquisition by intercropped faba bean and maize (*Zea mays* L.) increased by 20% when no P was supplied and by 38% when P was applied at 33 kg ha^−1^ 11.

Rhizosphere processes facilitate P uptake in cereal/legume intercropping^6^,^7^,^12^,^13^,^14^. For example, intercropped faba bean, compared with maize, has a much stronger ability of rhizosphere acidification, which increases soil P^15^. In agar gel, faba bean decreases its rhizosphere pH by approximately 2 units in 6 hours^10^. Intercropped chickpea (*Cicer arietinum* L.) secretes acid phosphatase into the rhizosphere, thus mobilizing organic P and increasing P acquisition of the neighboring maize^7^. White lupin (*Lupinus albus* L.) exudes a large amount of citrate to mobilize sparingly soluble P in soil that is not normally available to wheat^16^. Concordantly, Li, *et al.*^6^ have found that wheat intercropped with common bean (*Phaseolus vulgaris* L.) exhibits a biomass increase, resulting from the alleviated competition caused by tapping different P fractions. In general, legumes can mobilize sparingly soluble P, from which the neighboring cereals can also benefit, resulting in an overall increase in P uptake by cereal/legume intercrops^17^. However, many of these studies have obtained this general picture only by comparing the differences of biomass and P content between intercropped crops from limited harvests or the final harvest. Therefore, the earlier or unmeasured processes contributed to plant interactions between intercropped species might be neglected.

Recent studies^18^,^19^,^20^ have characterized the dynamic process of competitive nitrogen (N) uptake by isolated and mixed plants using a logistic model. Those dynamic processes indicated that competition does not always dominate during the whole growth period and that plant competition changes to facilitation at some time point at the latter growth stage. Unfortunately, the visual dynamics of P uptake by plants and rhizosphere processes are lacking, compared with the well-documented N facilitation in cereal/legume intercropping^21^. Therefore, it is necessary to provide experimental evidence of the daily dynamics of P uptake between intercropped plants and to describe the rhizosphere processes contributing to facilitative P uptake when wheat is planted together with faba bean. The objectives were to visualize the dynamics of biomass accumulation and the P uptake of isolated, monocropped and intercropped wheat in an additive design, and to determine when and how rhizosphere processes that were altered by faba bean increase P uptake of intercropped wheat.

## Results

### Plant growth and phosphorus content

**Figure 1:**
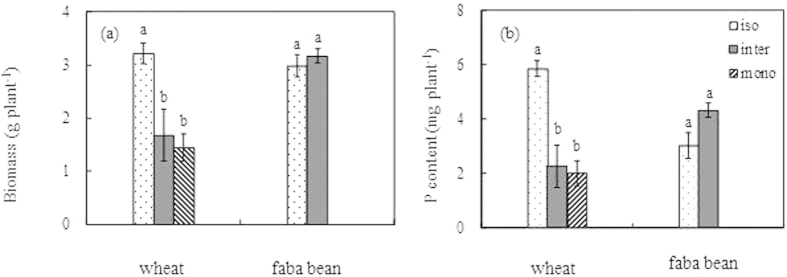
The biomass (a) and P content (b) of wheat and faba bean in the isolated (iso), monocropped (mono) and intercropped (inter) conditions at the final harvest. Different letters above the bars indicate the significance under *P* ≤ 0.05. The error bars indicate the standard error (SE).

The biomass of wheat plants at the final harvest when grown isolated was 3.22 g dw plant^-1^. When grown as a monocrop, with two neighbors, the biomass decreased significantly by 48% to 1.68 g dw plant^-1^ due to intraspecific competition (Fig. 1a). The final biomass did not further decline when wheat was intercropped with one faba bean plant. Additionally, at final harvest, the biomass of isolated faba bean plants did not differ from that of plants grown together with three wheat plants.

The plant P content responded similarly to the biomass between different treatments. The phosphorus content of monocropped wheat and intercropped wheat was 61% and 72%, respectively, lower than that of isolated wheat. The difference in P content between intercropped and isolated faba bean was not significant (Fig. 1b)

**Figure 2:**
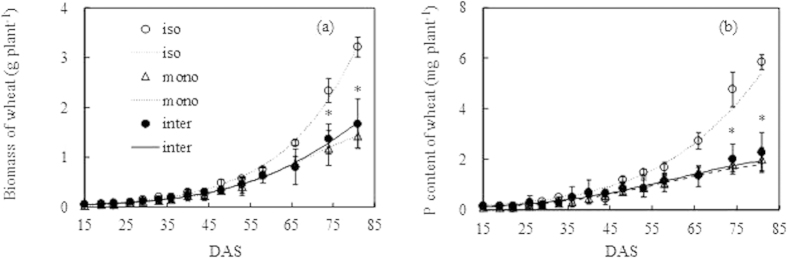
The cumulative biomass production (a) and P content (b) of wheat in isolated (iso), monocropped (mono) and intercropped (inter) conditions. The symbols indicate the measured biomass and plant P content at each harvest. The values indicate the means ± SE, n = 3. The curves indicate the cumulative biomass production and P content derived from the logistic model. The asterisks indicate that the increments of biomass and P content of intercropped wheat from 66 DAS to 74 and 81 DAS were significantly higher than the corresponding increment of monocropped wheat during these two periods.

The cumulative biomass production curves of isolated wheat did not differ from that of monocropped and intercropped wheat until 40 DAS (Fig. 2a). The cumulative biomass production curves of monocropped wheat diverged from intercropped wheat only after 66 DAS. The biomass increment of intercropped wheat from 66 to 74 and to 81 DAS was significantly higher (*P* < *0.05*) compared with the corresponding increment of monocropped wheat during these two time intervals. Intercropped wheat increased 1.69 g plant^-1^ and 2.16 g plant^-1^ from 66 DAS to 74 and to 81 DAS, respectively, which were 1.9 and 1.3 times the increment of monocropped wheat during the same intervals. The trend of the cumulative P uptake curves was similar to that of the biomass production. The P content of the intercropped wheat significantly increased (*P* < *0.05*) compared with that of monocropped wheat after 66 DAS, in line with the results of the cumulative biomass production (Fig. 2b). The phosphorus content of intercropped wheat increased 2.00 mg plant^-1^ and 2.79 mg plant^-1^ at 74 DAS and 81 DAS compared with that at 66 DAS, which were 2.3 and 1.9 times the increment of monocropped wheat during these two time intervals.

**Figure 3:**
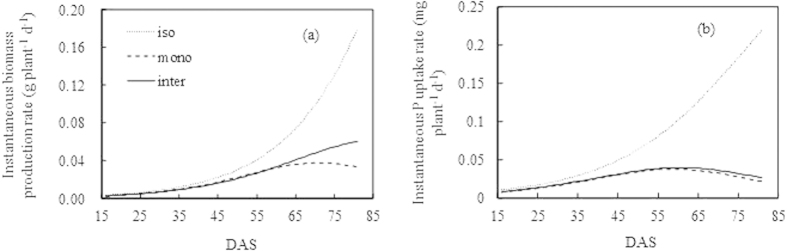
The instantaneous biomass production rate (a) and P uptake rate (b) of wheat in the isolated (iso), monocropped (mono) and intercropped (inter) conditions by calculating the slopes of the cumulative biomass production curves (Fig. 3a) and P uptake curves (Fig. 3b).

The isolated wheat produced biomass at a rapid rate, and the rate sharply increased at the end of the experiment (Fig. 3a). The rate was 0.18 g plant^-1^ d^−1^. With the presence of neighboring wheat plants, the instantaneous biomass production rate of monocropped wheat maintained a slow increase, and the maximum rate was only 0.03 g plant^-1^ d^−1^ at harvest. However, when intercropped with faba bean, wheat produced biomass much rapidly than monocropped wheat after 58 DAS and reached 0.06 g plant^-1 ^d^−1^ at final harvest, which corresponded to the significant biomass increment of intercropped wheat in Fig. 2a.

The instantaneous P uptake of isolated wheat followed a similar trend as the instantaneous biomass production rate, and the maximum instantaneous P uptake rate was 0.22 mg plant^-1^ d^−1^ at harvest (Fig. 3b). However, with intraspecific and interspecific competition, the instantaneous P uptake rate by wheat showed a unimodal pattern. Monocropped wheat attained its maximum instantaneous P uptake rate (0.04 mg plant^-1^ d^−1^) at 59 DAS, whereas the intercropped wheat reached that (0.04 mg plant^-1^ d^−1^) at 60 DAS.

### Plant phosphorus concentration

**Figure 4:**
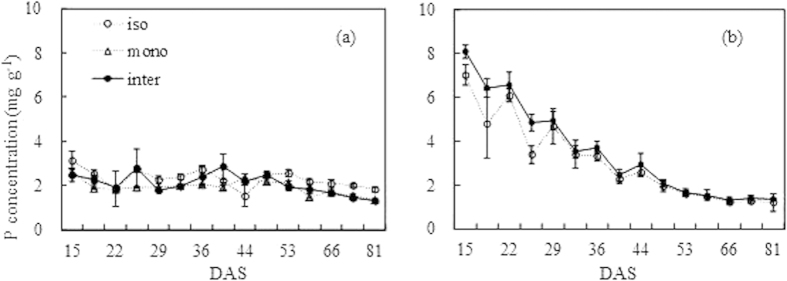
Phosphorus concentration of wheat (a) and faba bean (b) in the isolated (iso), monocropped (mono) and intercropped (inter) conditions. The bars indicate the standard error of three replicates.

The P concentration of shoots showed a decreasing trend for both wheat and faba bean. The P concentration of isolated wheat decreased from 3.14 to 1.82 mg g^−1^, and it was approximately 2 mg g^−1^ for most of the growing time (Fig. 4a). The P concentration of monocropped wheat decreased from 2.64 to 1.38 mg g^−1^, and was lower than 2 mg g^−1^ after 58 DAS. For intercropped wheat, the P concentration fluctuated between 2.86 and 1.34 mg g^−1^, and was lower than 2 mg g^−1 ^after 53 DAS. The P concentration of isolated wheat was significantly higher (*P* < *0.05*) than that of monocropped and intercropped wheat at some sampling dates, especially after 53 DAS.

The descending trend of faba bean was more obvious than that of wheat (Fig. 4b). Isolated faba bean had a decreasing P concentration from 7.02 to 1.20 mg g^−1^, which was lower than 2 mg g^−1^ after 48 DAS. In intercropped faba bean, similarly to isolated faba bean, the P concentration decreased from 8.09 to 1.36 mg g^−1^, and was lower than 2 mg g^−1^ after 53 DAS.

### Carboxylates in rhizosphere

**Figure 5:**
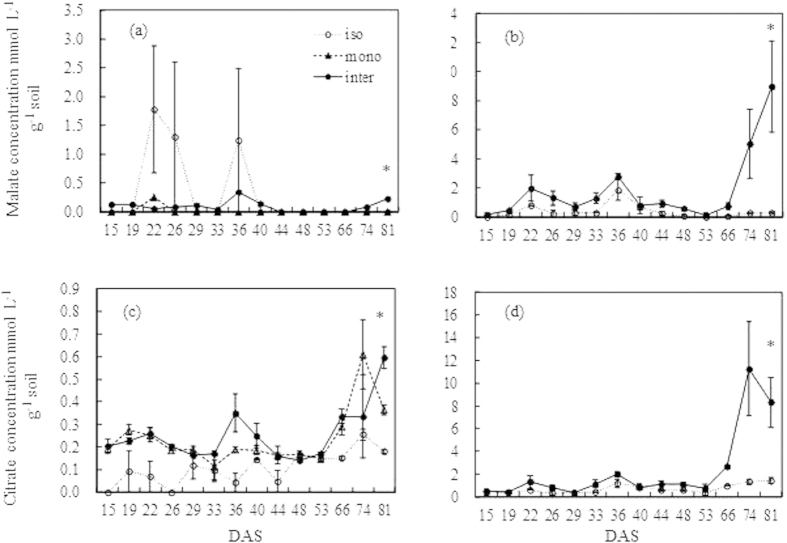
Malate and citrate concentrations in the rhizosphere soil of wheat (a, c) and faba bean (b, d) in isolated (iso), monocropped (mono) and intercropped (inter) conditions. The asterisks indicate that the difference was significant. The error bars indicate the standard error (SE). The data in 58 DAS was unavailable.

Several kinds of carboxylates including malate (Fig. 5a,b), citrate (Fig. 5c,d), and succinate, fumarate and T-aconitate at low concentrations (data not shown) were found in the rhizosphere soils of wheat and faba bean. The isolated and monocropped wheat roots secreted minimal amounts of malate during the whole growth stage. At the final harvest, intercropped wheat rhizospheres had significantly more malate than isolated and monocropped wheat.

The malate content in the rhizosphere of faba bean was higher than in wheat in each harvest (Fig. 5b). Although it fluctuated in time, the increment of the malate content of intercropped faba bean at later harvest times was much greater, and was 10.8, 18.6 and 29.4 times that of isolated faba bean at 66, 74 and 81 DAS, respectively. The rhizosphere of monocropped and intercropped wheat had a higher content of citrate than that of isolated wheat, but the amount of citrate in the all wheat rhizosphere showed a growing trend especially at the end of experiment. Similarly to the variation of the malate concentration in the faba bean rhizosphere, the citrate content in the rhizosphere of both isolated and intercropped faba bean increased, and the increment of the intercropped faba bean was significantly higher (*P* < *0.05*) than that of isolated faba bean.

### Acid phosphatase activity in the rhizosphere

**Figure 6:**
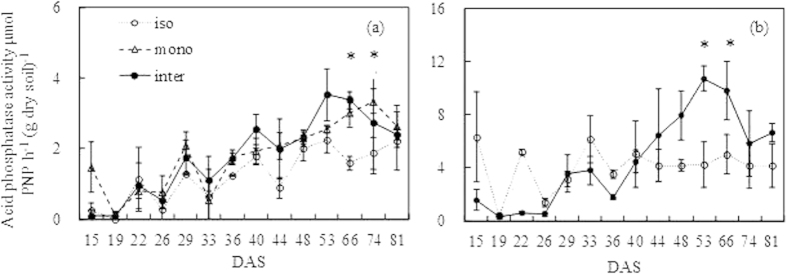
Rhizosphere acid phosphatase of wheat (a) and faba bean (b) in the isolated (iso), monocropped (mono) and intercropped (inter) conditions. The asterisks indicate that the difference was significant. The error bars indicate the standard error (SE). The data in 58 DAS was unavailable.

There was an overall increase in acid phosphatase activity in the wheat and faba bean rhizospheres over time, although it varies between harvests (Fig. 6). The acid phosphatase activity of intercropped wheat and monocropped wheat was significantly higher (*P* < *0.05*) than that of isolated wheat at 66 and 74 DAS (Fig. 6a). The phosphatase activity in the rhizosphere of intercropped faba bean was significantly higher (*P* < *0.05*) than that of wheat at the late growth period (after 40 DAS) (Fig. 6b). The acid phosphatase activity of the intercropped faba bean was higher than that of isolated faba bean at 53 and 66 DAS.

### Soil Olsen-P

**Figure 7:**
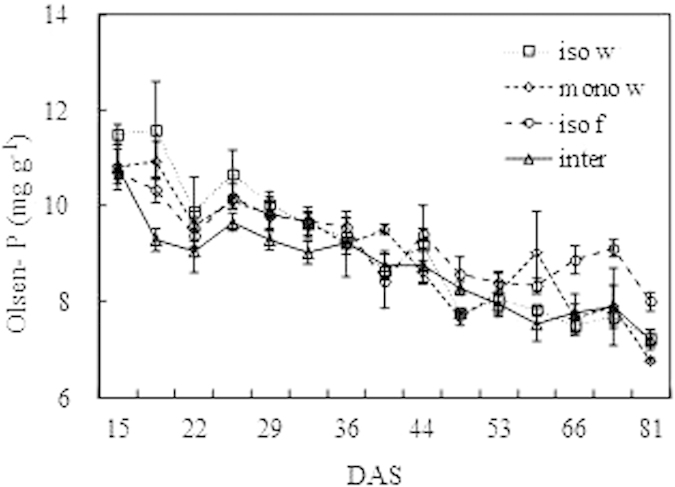
Soil Olsen-P of the different treatments. Iso w means the isolated wheat, iso f indicates the isolated faba bean, mono w means the monocropped wheat, inter means the intercropped wheat and faba bean. The error bars indicate the standard error (SE).

The soil Olsen-P of the bulk soil in all treatments decreased to approximately 7.3 mg kg^−1^ (Fig. 7). Overall, the Olsen-P of the intercropped treatments was lower than that of the isolated or monocropped treatments.

## Discussion

An additive design was used in this experiment, i.e., the density of intercropped wheat and faba bean is identical to that in the corresponding pure stand^22^. In the present study, the lack of an obvious difference in the cumulative biomass production and P uptake before 44 and 35 DAS between isolated and monocropped wheat indicated that the P supply was sufficient for plants growth, which was in accordance with results from previous studies^18^,^23^. With low-P soil and depleted available P, all plants in the present study should be P deficient, according to the low P concentration level after 44 DAS. The present study showed that monocropped wheat coexisted with faba bean at the early stage (until 65 DAS) and did not suffer interspecific competition by faba bean with respect to biomass production and P uptake. The strong intraspecific competition between wheat plants caused a reduction of biomass production and P uptake after 44 and 35 DAS, respectively, which reached 52% and 39% that of isolated wheat at the final harvest, respectively. However, no significant reduction of biomass and P uptake was caused by interspecific interactions. Weaker interspecific competition occurred between intercropped wheat and faba bean in the intercropping condition. The reason might be complementary effect between intercropped crops, which can utilize different forms of P sources or stimulate complementary microbial communities. As reviewed by Richardson^24^ that microorganisms can effectively increase root surface to access more soil P and also microbial biomass P constitutes to a large potential P pool for plants. But rhizosphere communities often affected by plant species, owing to the differential rhizodeposition and particularly root exudate amount and composition in different plant rhizospheres^25^. The microbial community structures of legumes were different from that of cereals, for instance, the bacterial community composition of chickpea differed from that of rape and Sudan grass because of its large exudation of organic acid^26^. Therefore, it is speculated that different microbiological diversity and function of wheat and faba bean might be complementary to alleviate the interspecific competition. Additionally, different species occupy their own resource niches^27^. Previous studies have revealed that wheat utilizes NaHCO_3_-and NaOH-extractable organic P in soil, the P utilization is different from that of common bean, thus helping to alleviate competition^6^. And compared with faba bean, wheat depleted less water-extractable inorganic P in the soil^28^. In the present study, during 44 to 74 DAS, there was a higher APase activity and a higher content of citrate in the rhizosphere of intercropped faba bean than that of wheat. These rhizosphere processes support P uptake of only the faba bean, and there is not enough extra mobilized P to facilitate P uptake of neighbored wheat. As explained by Casper and Jackson^29^, the reduced levels of soil resources have a negative impact on the performance of competing plants. In the present study, depleting soil available P caused reduced interspecific competition for biomass production and P uptake between intercropped wheat and faba bean. P-mobilization resulting from rhizopshere processes played an important role in the reduced competition.

The instantaneous rate of biomass production and P uptake calculated from the logistic model confirmed the cumulative results. The advantage of measuring instantaneous rates is that they can provide the best, unambiguous information about the actual competitive process^18^. However, the competition effect occurred 5–10 days earlier on the instantaneous rate of biomass production and P uptake than on the cumulative results. This difference is reasonable because the transformation from instantaneous to cumulative effects takes time. The intraspecific and interspecific interaction effect on the instantaneous rate of P uptake was also earlier than that of biomass production, which indicated that P was the growth-limiting factor at the end of the growth period. The limited soil P supply reduced the instantaneous rate of biomass production and P uptake. Nord and Lynch^30^ have suggested that the slow growth and phenological delay caused by low soil P supply is beneficial, because it permits more time for P acquisition and utilization. In the present study, this was the reason that the reduction of the P concentration of monocropped wheat was much less than the reduction of the instantaneous rate of P uptake compared with isolated wheat. The strong capacity of P mobilization allowed faba bean to avoid P uptake competition from the neighboring wheat, as documented by Li, *et al.*^10^.

Carboxylate exudation by roots plays an important role in the mobilization of the inorganic phosphate of soil when plants suffer P deficiency^31^, although the carboxylates exuded from roots have been detected in a considerable amount only in the first 4 mm of soil from the root surface^28^,^32^. In the present study, malate and citrate were the major carboxylates in the rhizosphere of faba bean and wheat, which was in agreement with the findings of Li, *et al.*^13^ and was also in line with the review by Hinsinger^2^, which described that among root exudates, citrate and malate are the most frequently involved in plant roots in response to P starvation. Here, the concentrations of carboxylates in the rhizosphere of faba bean were 10 or 20 times higher than that of wheat, indicating a strong capacity of soil P mobilization by faba bean. These differences were in accordance with previous studies in different species, showing a predominance of malonate in chickpea and citrate and malate in white lupin, whereas wheat exudes minimal amounts of these compounds^33^,^34^. For faba bean, the more P-efficient accessions had higher rhizosphere malate concentrations than the P-inefficient genotypes^35^. Compared with white lupin and field pea, faba bean exuded a lower amount of carboxylates, but the amount was greater than that in wheat, a species with slow exudation rates^36^. Therefore, when intercropped with faba bean, the amount of exudates of wheat was significantly higher than that of isolated or monocropped wheat during the later growth stage, because of the inevitable diffusion of carboxylates from the rhizosphere of the neighboring faba bean to the rhizosphere of intercropped wheat^37^.

The shoot P concentration of wheat remained at approximately 2 mg g^−1^, indicating that the P uptake rate of wheat follows the dry matter production^38^. However, the P concentration in faba bean decreased sharply from seedling stage to early-pod filling, which was a consequence of a dilution effect, as the biomass increased^6^. From 66 DAS, the shoot P concentration of faba bean was lower than 2 mg g^−1^, the critical level for biomass production of crops (e.g., maize and white lupin)^37^,^39^. Thereafter, the amount of rhizosphere carboxylates in intercropped wheat and faba bean increased significantly, because of the rhizosphere response of intercropped wheat and neighboring faba bean to P depletion.

The P deficiency stimulated secretion of acid phosphatase from the roots of various plant species^40^,^41^. For example, a significant positive correlation was observed between the depletion of organic P and phosphatase activity in the rhizosphere soil of wheat and clover^42^. Plant roots with a higher phosphatase activity have a greater potential to utilize soil organic phosphorus^43^. In the present study, faba bean root secreted acid phosphatase with higher activity than wheat, and the acid phosphatase activity increased with growing time, which was accordance with the results of Tarafdar and Jungk^42^. The species variation in acid phosphatase secretion and facilitative utilization of P confirmed previous reports^7^,^12^. Li, *et al.*^7^ have observed that chickpea with higher acid phosphatase activity can mobilize organic P to improve total P uptake by intercropped maize. Moreover, with an organic P source (phytate) supply, P uptake by intercropped wheat was facilitated by chickpea^12^. In this experiment, another control of monocropped four wheat plants might be included. This control could be further explained the effect of adding one faba bean plant to monocropped wheat compared with adding one wheat plant, because faba bean has much stronger rhizosphere alteration ability than wheat.

The results suggest that intercropped faba bean with increased exudation of carboxylates and higher acid phosphatase activity mobilized P at the later growth stage. However, the amount of mobilized P might not be enough for intercropped wheat. Therefore, the biomass and P content of intercropped wheat were not significantly higher than that of monocropped wheat, which was similar to the results of Betencourt, *et al.*^14^. One reason might be that the growth period was not long enough because of the slow growth rates of wheat and faba bean. However, on the basis of the significant increment of the biomass and P content of intercropped wheat compared with that of monocropped wheat at the final two samplings, facilitation might occur and facilitation could be verified with a longer growth period.

## Conclusions

The logistic model visualized the dynamic growth pattern and P uptake of wheat with neighboring faba bean according to depletion of available P in the soil. Soil P supply was the major factor used to determine the interaction between wheat and faba bean grown in a P-limited intercropping system. In the late growth stage, carboxylate exudation by intercropped faba bean with P deficiency seemed resulted in facilitation of the P uptake of intercropped wheat, although the facilitation was reflected by only the instantaneous biomass accumulation. The results showed that intercropping of wheat and faba bean resulted in efficient P utilization due to complementary P uptake behavior and positive interaction of the rhizosphere processes when soil P was depleted.

## Methods

### Experimental design

A pot experiment was executed with wheat (*Triticum aestivum* L. cv. Yunmai-42) and faba bean (*Vicia faba* L. cv. Yundou 324) plants. Each pot (260 × 170 mm) was filled with 2 kg of air-dried and sieved (2 mm) soil. The five treatments were isolated wheat or faba bean (1W; 1F) plants, monocropped wheat (3 wheat plants; 3W) and three wheat plants intercropped with one faba bean plant (3W/1F) and without plants (control). The ratio of intercropped plants was derived from the conventional practice^44^. Plants were thinned to the appropriate density for the four treatments 11 days after sowing (DAS). Treatments were replicated 3 times and there were 45 pots for each treatment to allow for 15 destructive samplings during the whole experimental period.

The soil was a loam collected from fallow land of the Shangzhuang Experimental Station, Beijing, China, and contained an organic carbon concentration of 10.1 g kg^−1^, a total N concentration of 0.6 g kg^−1^, a mineral N concentration (Nmin) of 18.3 mg kg^−1^: nitrate N, 15.1 mg kg^−1^ and ammonium N, 3.2 mg kg^−1^, Olsen-P of 11 mg kg^−1^, NH_4_Ac-exchangeable K of 74.1 mg kg^−1^, and a pH in CaCl_2_ of 7.8 (the ratio of soil to CaCl_2_ solution was 1:2.5). Basal nutrients were added to the soil as a solution (mg kg^−1^ soil): N, 200 as Ca(NO_3_)_2_, P, 0, K, 200 as K_2_SO_4_. The added nutrients were thoroughly mixed with the soil by shaking after the soil was air-dried.

The experiment was conducted from August to December 2012 in a glasshouse at China Agricultural University, Beijing, China. Air temperatures ranged from 13 °C to 25 °C. Evaporative cooling and shade cloth were used to prevent excessively high temperatures on sunny days. The pots were watered regularly to 70% of field capacity (21%, w/w). Window screens were used to prevent one plant from over-topping the other to avoid competition for light.

### Harvesting and data collection

A total of 15 destructive harvests were performed every 4–6 days. At each harvest, shoots were removed by cutting at the soil surface, and roots were removed from the soil carefully. The roots with tightly adhering rhizosphere soil were immersed in 50 mL 0.2 mM CaCl_2_ solution and shaken carefully to collect the rhizosphere solution. A subsample of the rhizosphere extract was stored at −20 °C for HPLC analysis^13^, whereas the other subsample was stored at 4 °C for determination of acid phosphatase activity. Finally, the remaining rhizosphere solution was air-dried and used as the rhizosphere soil weigh for the calculations. All harvested shoots were oven-dried at 76 °C for 72 h before weighing. The phosphorus concentration of plant shoots was determined using the vanodomolybdate method^45^ after wet digestion with a mixture of concentrated H_2_SO_4_ and H_2_O_2_.

The determination of the acid phosphatase activity of the intact roots was performed as previously described by Alvey, *et al.*^46^. The analysis involved colorimetric estimation of the p-nitrophenol released by phosphatase activity after incubation of soil with 4 mL of 0.04 M sodium maleate buffer (pH 5.3) at 28 °C for 30 minutes. The reaction was stopped by 0.5 M NaOH, and the absorbance was measured spectrophotometrically at 405 nm. One unit of acid phosphatase activity was defined as the activity per gram soil that produced 1 μmol p-nitrophenol per hour.

### Data analysis

The biomass (*Y*; mg) of one herbaceous plant grown from seed for several weeks is typically a sigmoid function of time, *t*. For each daily interval, Δ_*t*_, Y was computed using a discrete two-parameter form of the logistic equation^18^


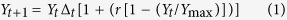


(*r*, the rate constant (d^−1^) for biomass production; *Y*_*max*_, a maximum to which *Y* tends asymptotically), which was used to separately fit the data of the shoot and root biomass and N content by simultaneously adjusting *r* and *Y*_*max*_ to maximize *R*^*2*^ with the SOLVER in Microsoft Excel 2010^47^. The instantaneous rates were derived as the slopes of the logistic models fitted to the cumulative biomass or N content data, rather than directly from the data.

One-way analysis of variance was performed on all datasets and treatments using the SAS statistical software (SAS, 2001). The mean differences of different treatments among species were determined based on the least significant difference (LSD) at the *P* ≤ 0.05 probability level.

## Additional Information

**How to cite this article**: Li, C. *et al.* Shift from complementarity to facilitation on P uptake by intercropped wheat neighboring with faba bean when available soil P is depleted. *Sci. Rep.* **6**, 18663; doi: 10.1038/srep18663 (2016).
